# Universal Supercritical Thermodynamics for Black Holes

**DOI:** 10.34133/research.1406

**Published:** 2026-08-28

**Authors:** Shoucheng Wang, Xinyang Li, Yuliang Jin, Li Li

**Affiliations:** ^1^ School of Science, Hunan Institute of Technology, Hengyang 421002, China.; ^2^ Institute of Theoretical Physics, Chinese Academy of Sciences, Beijing 100190, China.; ^3^ Peng Huanwu Collaborative Center for Research and Education, Beihang University, Beijing 100191, China.; ^4^ School of Physical Sciences, University of Chinese Academy of Sciences, Beijing 100049, China.; ^5^ Wenzhou Institute, University of Chinese Academy of Sciences, Wenzhou 325000, China.; ^6^ School of Fundamental Physics and Mathematical Sciences, Hangzhou Institute for Advanced Study, UCAS, Hangzhou 310024, China.

## Abstract

We investigate thermodynamic crossovers for black holes in the supercritical regime beyond the critical point, where small and large black holes become indistinguishable from the conventional viewpoint. We establish a refined supercritical phase diagram that comprehensively characterizes the phases of small, large, and indistinguishable black holes, delineated by 2 supercritical crossover lines. The universal scaling laws of these crossover lines are fully verified using the thermodynamics of Reissner–Nordström-anti-de Sitter black holes in both the standard framework and the extended thermodynamic phase space, where the cosmological constant is treated as pressure, as well as in 4 other black hole systems. Analogies with supercritical crossovers observed in liquid–gas and liquid–liquid phase transitions are discussed. This work can be extended to more complex black hole backgrounds and offers valuable insights into the fundamental nature of black hole thermodynamics.

## Introduction

Black hole thermodynamics extends classical concepts to extreme gravitational systems, revealing rich phase structures and critical phenomena. The interplay between geometry, thermodynamics, and holography underscores deep connections between gravity and statistical physics, with critical exponents universal across diverse systems. Of particular interest is the discovery of the charged Reissner-Nordström (RN) black hole in the anti-de Sitter (AdS) spacetime with a negative cosmological constant Λ. This black hole admits a first-order small-black-hole/large-black-hole (SBH/LBH) phase transition, which is in many respects analogous to the liquid–gas phase transition (LGPT) [1,2]. This analogy has triggered broad interest and stimulated numerous studies on the critical behavior of black holes, greatly enriching the phase structure of black holes, e.g., black hole chemistry [3–5] and quantum chromodynamics-like black hole phase [6–11]. In particular, by treating Λ and its conjugate quantity as thermodynamic variables associated with the pressure and volume, the RN-AdS black hole in this extended phase space displays classical critical behavior and is superficially analogous to the van der Waals LGPT. In contrast, in the nonextended phase space with Λ a fixed model parameter, the above analogy has not been well established, mainly due to the absence of good identification of pressure [2].

Although black hole critical phenomena have been extensively studied for over 2 decades since [1], little progress has been made in exploring the properties of black holes above the critical point, and the thermodynamic nature of supercritical black holes (SCBHs) remains unknown. The behavior in the supercritical region is fundamentally different from that below the critical point. For the latter, thermodynamic response functions change discontinuously when crossing the coexistence line. For SCBHs, the thermodynamic crossover curve (Widom line) was constructed via the Ruppeiner geometry [12,13], Lee–Yang zeros [14], kinetic crossover [15], and maximal scaled variance [16]. On the other hand, the supercritical dynamic crossover curve (Frenkel line) has been identified by transitions between distinct quasi-normal modes [16]. However, these supercritical crossover lines do not exhibit universal behavior.

In this work, we investigate 2 recently proposed thermodynamic crossover lines, *L*^±^ [17], which serve as boundaries of the SCBH region in the phase diagram. The *L*^+^ and *L*^−^ lines represent the loci where the thermodynamic response starts to deviate from LBH-like and SBH-like behavior, respectively. The region between the 2 lines is where the system no longer clearly resembles either phase, justifying the term “indistinguishable”. Our primary goal is to extend the *L*^±^ to black hole thermodynamics, which has lacked a systematic supercritical description. Unlike existing supercritical diagnostics (e.g., Widom or Frenkel lines) that do not show universal behavior in black holes, the *L*^±^ lines yield robust universal scaling laws, making them a natural tool for this purpose. Beyond universality verification, we envisage the scaling laws as a probe for beyond mean-field criticality in future studies.

We establish universal scaling laws for the *L*^±^ lines near 7 critical points in the following 6 black hole systems: (a) 4-dimensional (4D) RN-AdS black holes in the extended phase space, (b) 4D RN-AdS black holes in the nonextended phase space, (c) hairy black holes (with 2 critical points), (d) RN-AdS black holes in higher dimensions, (e) 5D charged Gauss–Bonnet black holes, and (f) Barrow black holes with fractal structure. We also discuss their analogies with LGPTs and liquid–liquid phase transitions (LLPTs).

## Results

### Comparison of various supercritical crossover lines for charged black holes in the extended phase space

**Fig. 1.**
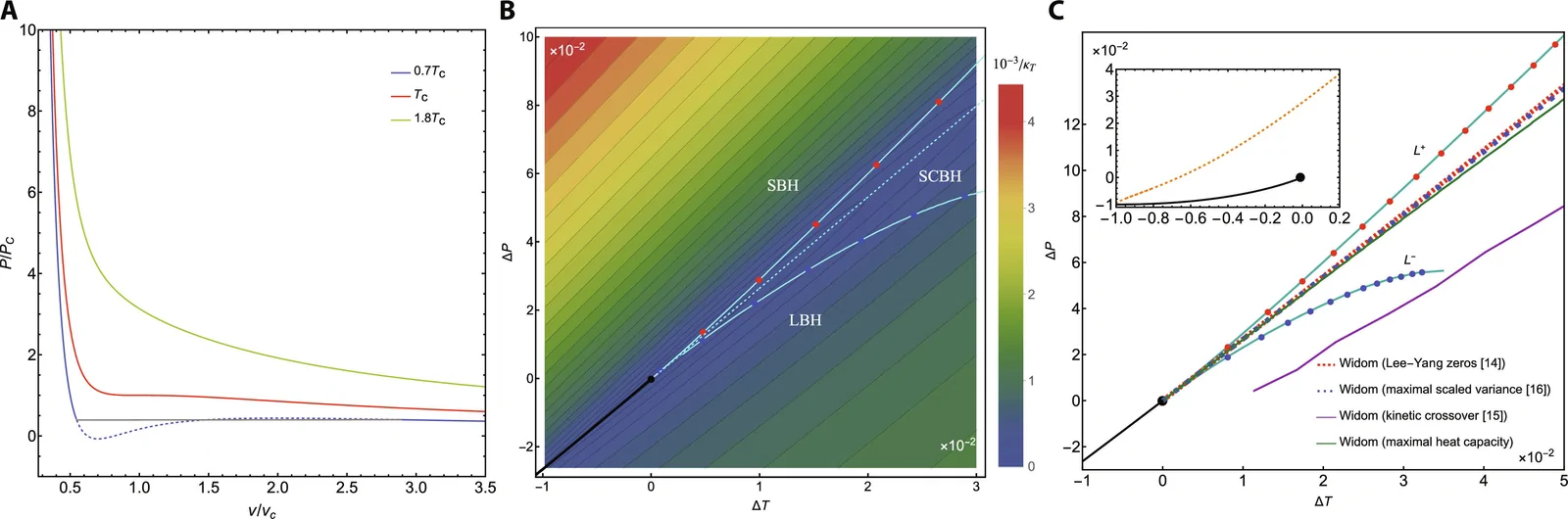
Supercritical thermodynamics for 4-dimensional Reissner-Nordström–anti-de Sitter black holes in the extended phase space. (A) Typical equations of state in 3 phases. Beyond the critical point $\left(T_{c},v_{c}\right)$ is the supercritical regime. (B) Phase diagram in terms of rescaled axes, $\Delta P=P/P_{c}-1$ and $\Delta T=T/T_{c}-1$. The solid black and dashed cyan lines represent respectively the coexistence line and critical isochore. The solid black point marks the critical point. The solid cyan line with red (blue) points represent the *L*^+^ (*L*^−^) line. The color map and contour lines are obtained via the isothermal compressibility. (C) Comparison with other supercritical crossover lines. The critical isochore is visually identical to the dashed Widom lines. The inset presents the Frenkel line (dashed) determined via quasi-normal modes [16]. We have set *Q* = 1.

We begin with the 4D RN-AdS black hole in the extended phase space. It has arguably the simplest thermodynamic properties, since its equation of state (EOS) remarkably coincides with the van der Waals EOS. The EOS is given as [2],

$$P\left(v,T\right)=\frac{T}{v}-\frac{1}{2\pi v^{2}}+\frac{2Q^{2}}{\pi v^{4}}\;,$$ (1)

where $P\equiv -\frac{1}{8\pi }\Lambda =\frac{3}{8\pi }\frac{1}{l^{2}}$ is the thermodynamic pressure (with *l* the AdS radius) [2], *v* the specific volume, and *Q* the charge. This black hole undergoes a small (liquid-like) to large (gas-like) black hole phase transition (see Fig. 1A). The critical point is located at $T_{c}=\frac{\sqrt{6}}{18\pi Q}$, $v_{c}=2\sqrt{6}Q$ and $P_{c}=\frac{1}{96\pi Q^{2}}$. Note that the critical compressibility factor $Z_{c}=P_{c}v_{c}/T_{c}=3/8$ in this black hole is identical to $Z_{c}=3/8$ in the van der Waals fluid. The inverse specific volume, ρ=1/v, has been identified as the number density of black hole molecules to measure the microscopic degrees of freedom [18]. The EOS Eq. (1) exhibits standard critical scalings near the critical point. For example, the isothermal compressibility scales as, ${\left.\kappa_{T}\equiv -\frac{1}{v}\frac{\partial v}{\partial P}\right|}_{T,Q}\propto {\left|T-T_{c}\right|}^{-\gamma }$, with *γ* =1, along the critical isochore ($v=v_{c}$). The order parameter $\rho_{g}-\rho_{l}$ ($\rho_{g}$ is the density of the gas-like phase and $\rho_{l}$ the liquid-like phase) on the coexistence line behaves as $\rho_{g}-\rho_{l}\propto {\left|T-T_{c}\right|}^{\beta }$, with β=1/2.

Li and Jin [17] proposed a new definition for supercritical crossover lines, named *L*^±^ lines, which account for the universal properties of critical points and the inherent symmetry of the phase diagram. These lines are operationally defined by locating the maxima of thermodynamic response functions along directions parallel to the critical isochore (the line of constant critical order parameter). Applying this definition to the RN-AdS black hole yields the *L*^±^ lines shown in Fig. 1B. These lines segment the black hole’s phase diagram into 3 characteristic zones: the SBH phase, a SCBH phase where SBH and LBH states are indistinguishable, and the LBH phase.

In addition to our work, alternative approaches have been proposed to analyze the supercritical properties of charged black holes, Eq. (1), which are summarized in Fig. 1C: The Widom line is a single crossover line defined by extrema of response functions [14–16]; the Frenkel line is also a single crossover line, which, however, does not originate from the critical point [16]; Ruppeiner geometry provides an alternative geometric method to locate the Widom line [18]; Lee–Yang zeros yield the Widom line via complex analytic continuation [14]; and kinetic crossover lines are dynamical in nature and often do not emanate from the critical point [15]. In contrast to all these existing crossover lines, the *L*^±^ construction provides a 2-line framework that divides the supercritical region into 3 regimes (SBH, indistinguishable, and LBH) and obeys universal scaling laws not shared by single-line diagnostics. Its limitations include the need for a suitable order parameter (which is not obvious in the nonextended phase spaces), its thermodynamic (rather than dynamical) character, and weak dependence on the choice of the response function away from the critical point.

### Universal scaling of *L*^±^ lines for 7 critical points in charged black holes

It has been suggested that *L*^±^ lines should follow universal scaling laws [17]:

$$\delta P^{\pm }\propto {\left(T-T_{c}\right)}^{\beta +\gamma }\;,$$ (2)

and

$${\delta \rho }^{\pm }\propto {\left(T-T_{c}\right)}^{\beta }\;,$$ (3)

near the critical temperature $T_{c}$. Here, *T* is the control field, ρ the order parameter, *P* the conjugated external field (ordering field), $\delta \rho^{\pm }$ ($\delta P^{\pm }$) measures the difference of ρ (*P*) between the *L*^±^ lines and the critical isochore line ($\rho =\rho_{c}$ line), and β,γ standard critical exponents whose values depend on the universality class. While these scaling relations have been examined in several nongravitational systems [17,19,20], it remains an open question whether gravitational effects preserve or alter this universality. A particularly crucial test would be applying these scalings to black hole systems that lack a clear van der Waals fluid analogy, where the persistence of universal behavior is uncertain.

**Fig. 2.**
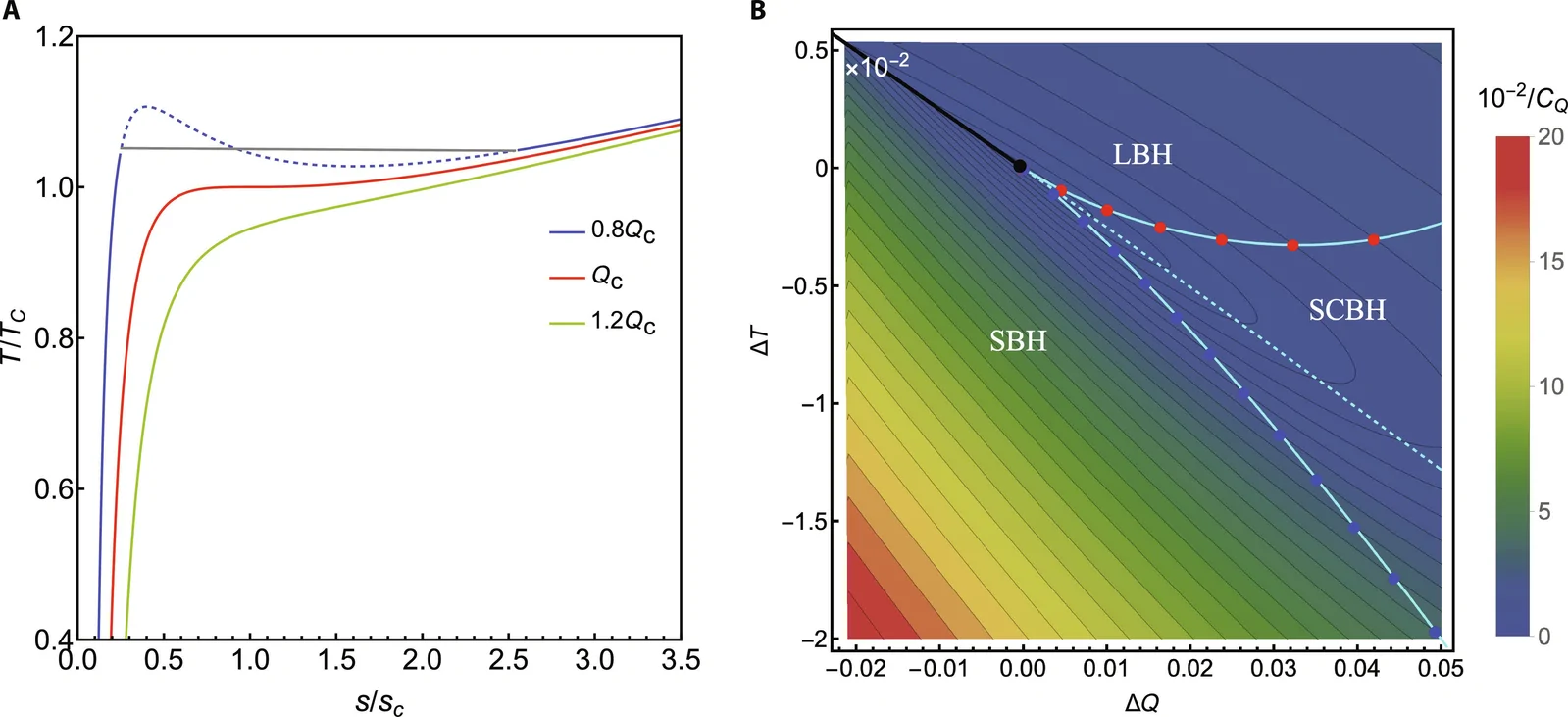
Supercritical thermodynamics for 4-dimensional Reissner-Nordström–anti-de Sitter black holes in the nonextended phase space. (A) Typical equations of state in 3 phases. Beyond the critical point $\left(T_{c},Q_{c}\right)$ is the supercritical regime. (B) Phase diagram in terms of rescaled *Q* and *T*. The solid black and dashed cyan lines represent the coexistence line and the critical isentrope $S=S_{c}$, respectively; the latter serves as the supercritical extension of the coexistence line. The solid cyan line with red (blue) points marks the *L*^+^ (*L*^−^) line. The color map and contour lines are obtained according to the specific heat. We have set $l=\sqrt{3}$.

To address this question, we analyze the supercritical thermodynamics of 7 distinct critical points in 6 black hole models (see Supplementary Materials Section S1 for details). These models span a broad range of physical settings and phase structures, including cases with non-van der Waals behavior. Remarkably, despite the introduction of nontrivial features such as quantum effects and fractal structures, the scaling relations defined in Eqs. (2) and (3) are universally observed. This finding suggests that the proposed scalings possess a robustness that transcends both specific gravitational models and the van der Waals analogy. The studied systems include the following black holes:

1. Charged black holes in the extended phase space (RN-AdS_4_), as detailed above.

2. Charged black holes in the nonextended phase space [RN-AdS_4_(NE)]. For such black holes, where Λ is a fixed model parameter, their correspondence to the liquid–gas system is debatable [2]. Nevertheless, a first-order SBH/LBH phase transition for the 4D RN-AdS black holes exhibits in the fixed charge ensemble (canonical ensemble) [1]; see the $\left(T,Q\right)$ phase diagram in Fig. 2A, where the first-order line is denoted by a solid black line with the critical point located at $\left(T_{c}=\frac{2}{\pi l\sqrt{6}},\mu_{c}=\frac{1}{\sqrt{6}},Q_{c}=\frac{l}{6}\right)$ .

In this case, the LGPT concepts of thermodynamic pressure and its conjugate order parameter (density) are no longer applicable. Alternatively, Kubiznak and Mann [2] and Chamblin et al. [21] proposed to treat *Q* as the control field and *T* as the ordering field. Following this idea, we identify the black hole entropy *S* (i.e., Bekenstein–Hawking entropy) as the order parameter conjugated to *T*; see also [22]. Along the coexistence line, the entropy gap between the 2 phases is $s=S_{2}-S_{1}$, which scales as $s\propto {\left|Q-Q_{c}\right|}^{\beta }$, with β=1/2. Along the critical isentrope ($S=S_{c}$), the response function ${\left.C_{Q}\equiv T\frac{\partial S}{\partial T}\right|}_{Q}$ follows the scaling $C_{Q}\propto {\left|T-T_{c}\right|}^{-\gamma }$ with γ=1. Following a similar procedure as explained in Methods, the *L*^±^ lines are determined (see Fig. 2B).

**Fig. 3.**
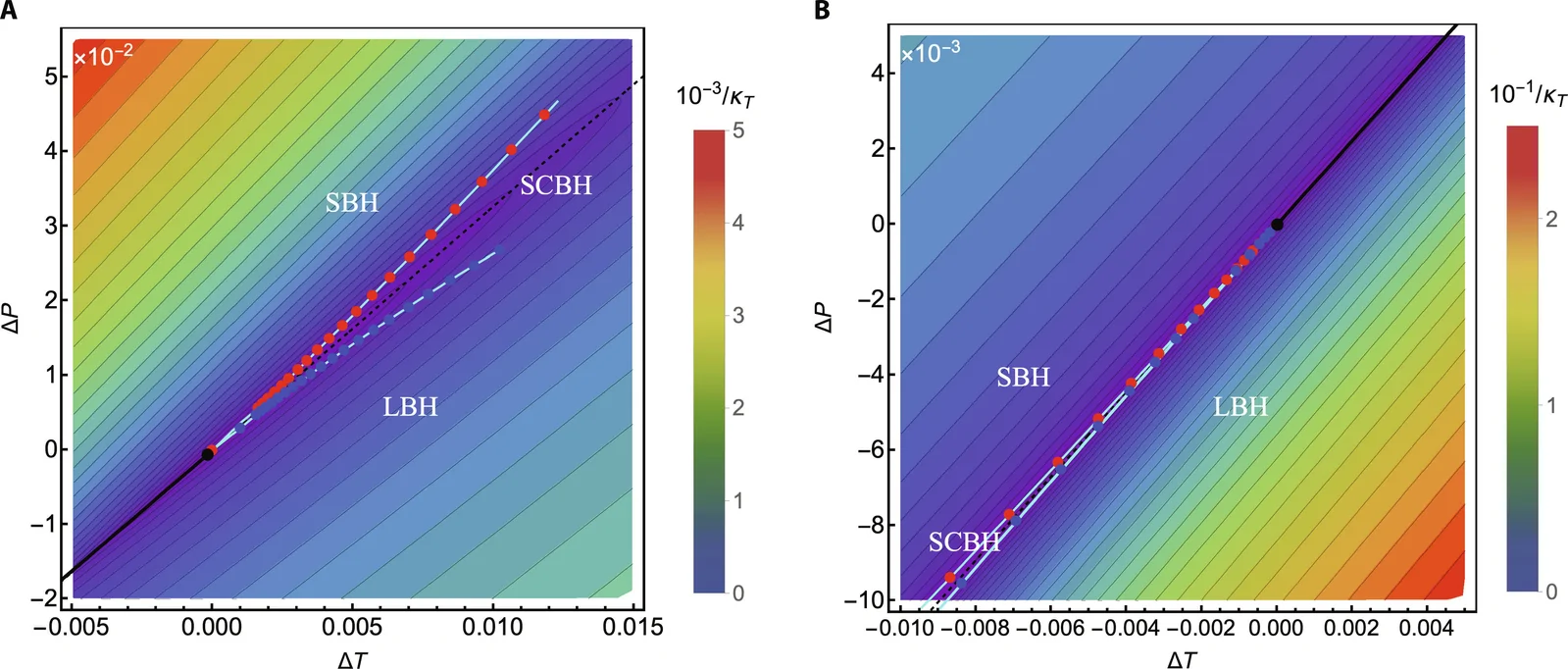
Supercritical thermodynamics for the hairy black hole [23], including (A) the Reissner-Nordström–anti-de Sitter-like critical point at large volume and low temperature and (B) another critical point at small volume and high temperature.

3. Hairy black holes with 2 critical points. The thermodynamics of a charged black hole with scalar hair in the extended phase space was studied in [23]. For a fixed charge *Q*, the system exhibits 2 critical points: one resembling the RN-AdS type at large volume and low temperature, and another at small volume and high temperature. As *Q* varies, the critical compressibility factor $Z_{c}=P_{c}v_{c}/T_{c}$ changes continuously, effectively tuning the nature of the dual fluid. This behavior clearly extends beyond the van der Waals description, even though the critical exponents *β* and *γ* at each critical point retain mean-field values. Following the same procedure outlined earlier for the RN-AdS black hole, we numerically determine the supercritical crossover lines *L*^±^, as shown in Fig. 3.

4. RN-AdS black hole in higher dimensions. The key thermodynamic features of the 4D RN-AdS black hole extend naturally to higher dimensions [24]. We find that the characteristic phase behavior, analogous to that shown in Fig. 1B, persists across dimensions.

5. Charged Gauss–Bonnet black holes. Higher-derivative curvature terms represent a common manifestation of quantum effects. Notably, the Gauss–Bonnet term arises naturally as a low-energy limit in string theory. Within the extended thermodynamic framework, we find that 5D charged Gauss–Bonnet black holes with spherical horizons [25] indeed possess supercritical crossover lines *L*^±^, analogous to those in Fig. 1B.

6. Barrow black holes with fractal structures. The Barrow model introduces a fractal-inspired modification to black hole entropy, expressed as $S_{B}=S^{1+\delta /2}$ [26] which generalizes the Bekenstein–Hawking entropy *S* to account for quantum gravitational spacetime foam. In this framework, the Barrow parameter *δ* (0≤δ≤1) measures the degree of quantum-gravitational deformation of the horizon area. Consistent with the first law, all other thermodynamic quantities are correspondingly rescaled by *δ*. When applied to the 4D RN-AdS black hole, the Barrow-modified thermodynamics shows supercritical crossover lines qualitatively similar to those in Fig. 1B. Notably, the conventional RN-AdS result is recovered in the limit *δ* = 0, illustrating how quantum-gravitational corrections (δ>0) alter—yet do not fundamentally disrupt—the observed thermodynamic pattern.

**Fig. 4.**
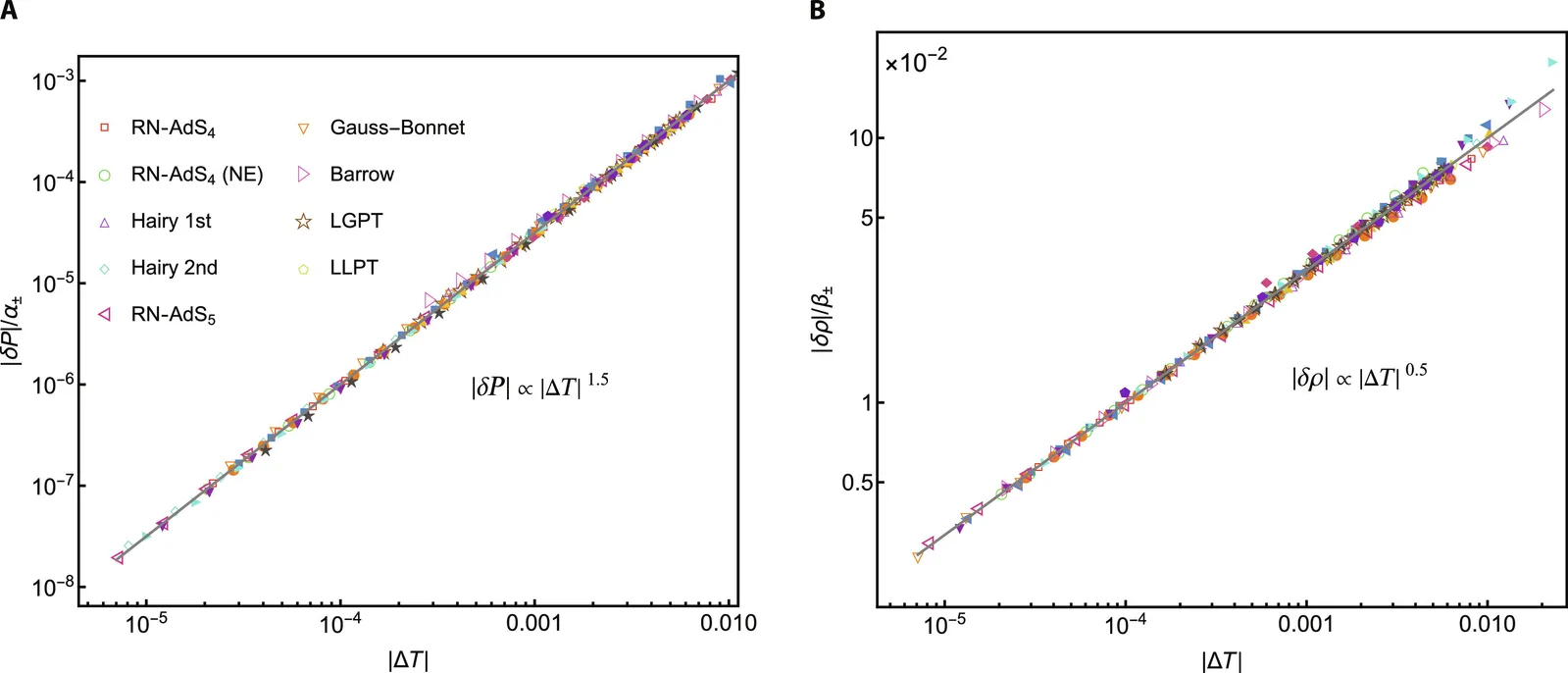
Scaling law near the critical point of (A) the external field, Eq. (2), and (B) the order parameter, Eq. (3), along the supercritical crossover lines, *L*^+^ and *L*^−^ (open and filled symbols correspondingly), for 6 black holes, the van der Waals liquid–gas phase transition (LGPT), and the liquid–liquid phase transition (LLPT) in the 2-state model. The values of *α*_±_ and *β*_±_ are summarized in Table 2.

Our numerical calculations confirm that near all critical points, the scaling of the *L*^±^ lines follows Eqs. (2) and (3). Although asymptotically exact only as $T\to T_{c}^{+}$, these scaling laws are found to hold effectively in a broad supercritical window for all systems studied, where higher-order corrections [27] remain negligible. This is illustrated in Fig. 4, which includes 14 supercritical scaling lines derived from 7 distinct black hole critical points (each critical point corresponds to 2 lines, *L*^±^). As summarized in Table 1, for the extended phase-space (E-BH), *P* is the external field and *ρ* is the order parameter. For the nonextended phase space (NE-BH), *T* serves as the external field, with *S* being the corresponding order parameter ($S_{B}$ in Barrow black holes). Note that other definitions of supercritical crossover lines, including Widom [15,28] and Frenkel lines [29,30], do not enjoy the universal scalings, Eqs. (2) and (3).

**Table 1.** Analogies between charged black holes (in the extended phase space [E-BH] and nonextended phase space [NE-BH]) and fluid systems (LGPT and LLPT)

Parameter	E-BH	NE-BH	LGPT	LLPT
Control field	*T*	*Q*	*T*	*P*
Ordering field	*P*	*T*	*P*	*T*
Order parameter	*ρ*	*S*	*ρ*	*S*

LGPT, liquid–gas phase transition; LLPT, liquid–liquid phase transition

### Analogous to LGPTs and LLPTs in fluids

The charged black hole EOSs in the extended thermodynamic phase space, given by Eq. (1), have been compared to the van der Waals EOS (see, e.g., [2]). This analogy is illustrated by comparing the EOSs and phase diagrams of black holes (Fig. 1A and B) with those of the LGPT in water (Fig. S2A and B). Extending this analogy to the supercritical regime, we find that both systems obey the same universal scaling laws along the *L*^±^ lines, as given by Eqs. (2) and (3) (see Fig. 4 for the LGPT given by the van der Waals EOS).

The correspondence of standard charged black holes in the nonextended phase space to the liquid–gas system has been debated [2]. Instead, here, we propose an analogy between the thermodynamics of these black holes and that of LLPTs. A LLPT is a first-order transition between low- and high-density liquid phases, terminated at a liquid–liquid critical point [31]. Comparing the EOSs and phase diagram of such black holes (Fig. 2A and B) with those of a typical LLPT (Fig. S2C and D) reveals 2 key similarities: (a) The coexistence line in the phase diagram has a negative slope—a characteristic feature of LLPTs [32]—in contrast to the positive slope found in LGPTs. (b) A natural order parameter is the entropy *S*, conjugate to the temperature *T*. Notably, it has been suggested that LLPTs are entropy-driven rather than energy-driven [33]. Coincidently, Kubiznak and Mann [2], Chamblin et al. [21], and Hosseini Mansoori et al. [22] have proposed treating *T* as the ordering field and *S* as the order parameter for charged black holes in the nonextended phase space. These correspondences are summarized in Table 1. We find that both black holes and LLPTs obey the same universal supercritical scaling laws given by Eqs. (2) and (3) (see Fig. 4). EOSs and phase diagrams of the LGPT and LLPT are summarized in Section S2.

## Conclusion

The main contribution of this work is the systematic extension of the *L*^±^ crossover lines to black hole thermodynamics. Unlike other supercritical diagnostics, they exhibit universal scaling behavior across diverse black hole models. Our findings not only underscore the universality of the supercritical thermodynamics for black holes but also reinforce the idea that supercritical phenomena near critical points are governed by universal principles, even in strongly curved spacetime geometries. We have numerically verified the universal scaling of the *L*^±^ crossover lines across a wide range of black hole models with diverse physical parameters and phase structures (Fig. 4). Although the critical exponents in the models studied here take mean-field values, our universality claim is not about the mean-field exponents themselves but rather about the fact that the *L*^±^ scalings hold over a finite supercritical window for the entire crossover lines across diverse gravitational models with drastically different phase structures, including cases beyond the van der Waals equation of state. This robustness is not guaranteed by standard near-critical mean-field theory.

Therefore, this leaves many interesting directions, including (a) generalization to complex black holes whose phase diagrams are with multiple critical points [34,35] and non-AdS black holes [36–40], (ii) extension to non-mean-field theories by incorporating quantum effects, such as those predicted by holographic duality or loop quantum gravity [22,41], and (c) exploration of supercritical dynamic crossover phenomena to bridge the gap between equilibrium and nonequilibrium criticality [20]. (d) Moreover, based on the celebrated holographic correspondence, generalization of the current approach can also be used to infer the quantum chromodynamics phase diagram [42]. (e) Finally, by further integrating tools from statistical mechanics and quantum field theory, future research may unravel deeper connections between black hole microstates and macroscopic phase structures, providing valuable insights into the fundamental nature of quantum gravity.

## Methods

### Determination of *L*^±^ supercritical crossover lines in charged black holes

We take the 4D charged RN-AdS black holes as examples to explain how to determine *L*^±^ lines. It can be straightforwardly generalized to other black holes.

**Fig. 5.**
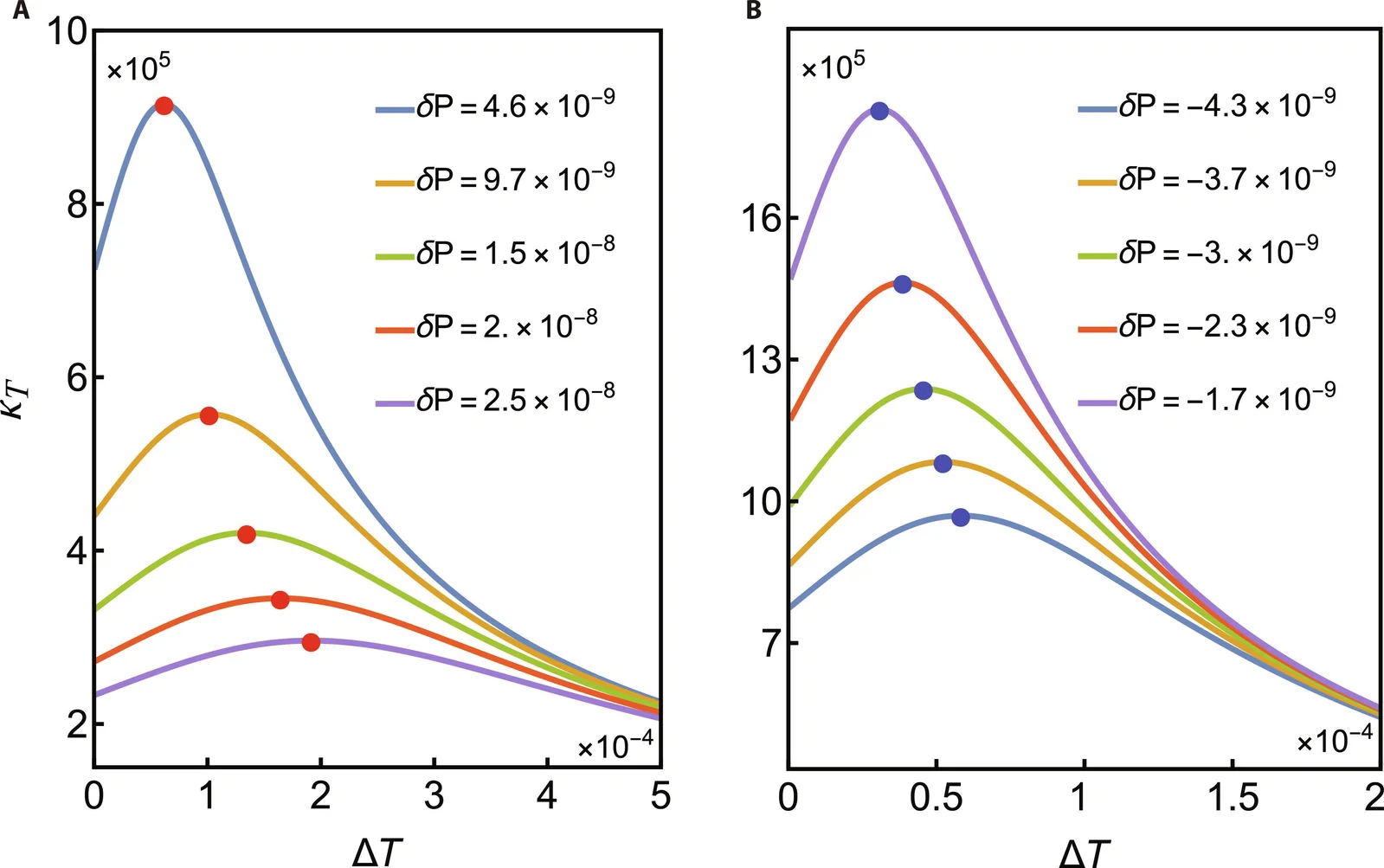
Supercritical crossover lines for the Reissner-Nordström–anti-de Sitter black holes in the framework of extended phase space. The susceptibility $\kappa_{T}$ is shown as a function of ΔT, for a few fixed (A) δP>0 and (B) δP<0. The peaks (red and blue dots) determine *L*^±^. We have set *Q* = 1.

We first focus on RN-AdS black hole in the extended phase space. To define the crossover lines proposed in [17], one first chooses the critical isochore as an extension of the coexistence line to the supercritical region. The compressibility $\kappa_{T}$ is evaluated along each path parallel to the critical isochore. Since $\kappa_{T}$ is a function of distance $\delta P\left(v,T\right)=P\left(v,T\right)-P\left(v_{c},T\right)$ and *T*, one can find a temperature $T_{\max}\left(\delta ,P\right)$ that maximizes $\kappa_{T}$ along each path. All the $T_{\max}\left(\delta ,P\right)$ points under different δP together consist of the thermodynamic crossover lines *L*^±^, on 2 sides of the critical isochore. Figure 5 depicts how the supercritical crossover lines *L*^±^ are determined for the charged black hole in Fig. 1. For a fixed δP, we find that $\kappa_{T}$ peaks at $T_{\max}^{+}\left(\delta ,P\right)$ for a given δP>0 (Fig. 5A) and $T_{\max}^{-}\left(\delta ,P\right)$ for a given δP<0 (Fig. 5B). The 2 resulting lines *L*^±^ are shown explicitly in Fig. 1B in the (ΔP,ΔT)-plane, where $\Delta P=P/P_{c}-1$ and $\Delta T=T/T_{c}-1$.

**Fig. 6.**
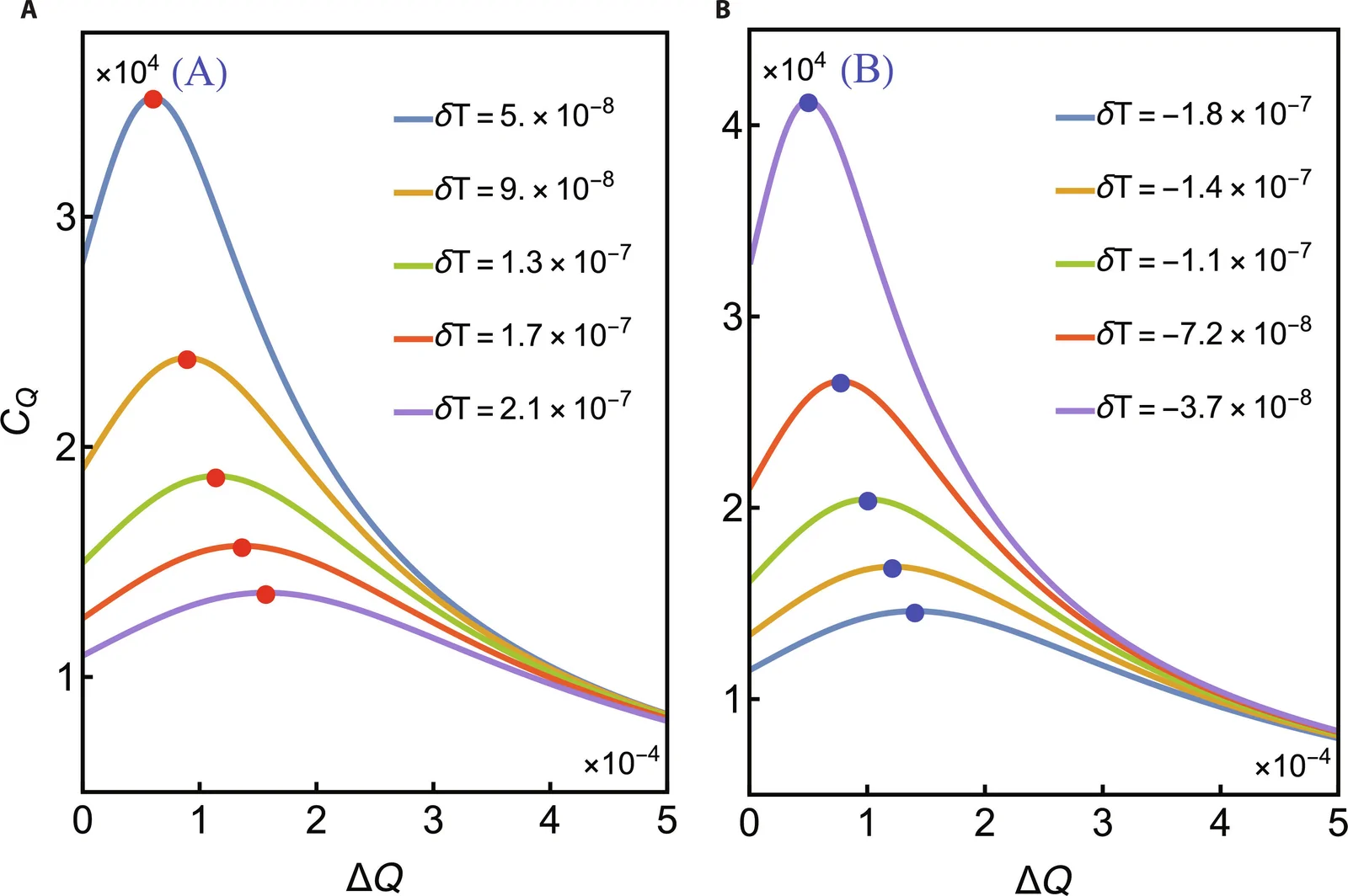
Supercritical crossover lines for the Reissner-Nordström–anti-de Sitter black holes in the nonextended phase space. The specific heat $C_{Q}$ as a function of ΔQ for a few fixed δT>0 (A) and δT<0 (B), respectively. The peaks (red and blue dots) correspond to supercritical crossover lines *L*^±^. We set $l=\sqrt{3}$.

For the case in the nonextended framework, we consider $C_{Q}$ as a function of $\Delta Q=Q/Q_{c}-1$ for a few fixed $\delta T\left(S,Q\right)=T\left(S,Q\right)-T\left(S_{c},Q\right)$. As shown in Fig. 6A and B, for a fixed δT, the specific heat $C_{Q}$ peaks at $Q_{\max}^{+}$ for δT>0 and peaks at $Q_{\max}^{-}$ for δT<0. We then obtain the supercritical crossover lines *L*^±^ shown explicitly in Fig. 2B.

### Coefficients of *L*^±^ lines

In Table 2, we list the coefficients $\alpha_{\pm }$ and $\beta_{\pm }$ obtained from the power-law fitting to the scaling forms, $\delta P^{\pm }=\alpha_{\pm }{\left(T-T_{c}\right)}^{\beta +\gamma }$ and ${\delta \rho }^{\pm }=\beta_{\pm }{\left(T-T_{c}\right)}^{\beta }$, for all systems in Fig. 4.

**Table 2.** Values of the coefficients *α*_±_ and *β*_±_

System	*α*_+_	*α*_−_	*β*_+_	*β*_−_
RN-AdS_4_	0.01	0.01	0.813	0.821
RN-AdS_4_ (NE)	0.106	0.105	1.43	1.39
LGPT	9.41	9.04	1.14	1.17
LLPT	0.004	0.005	0.177	0.183
Hairy 1st	0.075	0.094	1.08	1.12
Hairy 2nd	1.4	1.01	0.142	0.108
Barrow	0.006	0.008	0.73	0.958
RN-AdS_5_	0.038	0.046	0.581	0.701
Gauss–Bonnet	0.052	0.058	1.14	1.36

LGPT, liquid–gas phase transition; LLPT, liquid–liquid phase transition

## Data Availability

The data that support the findings of this study are available from the corresponding author upon reasonable request.

## Supplementary Materials

Supplementary 1 Sections S1 to S3 Figs. S1 to S3
