# Supplementary material for: Universal Supercritical Thermodynamics for Black Holes
Source: Research (Wash D C). 2026 Aug 28;9:1406. doi: 10.34133/research.1406 (PMC13522558; doi:10.34133/research.1406)
Supplement: Supplementary file 1 [file research.1406.f1.pdf]

SUPPLEMENTARY MATERIAL

Contents

S1 Black hole models **1**

S2 EOSs and phase diagrams of the LGPT and LLPT **7**

S3 Liquid-liquid phase transitions **8**

S1 Black hole models

We provide detailed description of the black hole models discussed in the main text. Except for 4D RN-AdS black holes, we consider the extended phase space in the canonical ensemble. The geometric units, $G_N = \hbar = c = k_B = 1$, are used.

A. The RN black hole in AdS$_4$

We begin with the spherical RN-AdS black hole in 4-dimensional spacetime from the Einstein-Maxwell action,

$$S_{EM} = \frac{1}{16\pi}\int d^4x\sqrt{-g}\,(R - F_{\mu\nu}F^{\mu\nu} - 2\Lambda)\;, \tag{S1}$$

where g is the determinant of the metric $g_{\mu\nu}$, R the corresponding Ricci scalar, $F_{\mu\nu} = \partial_\mu A_\nu - \partial_\nu A_\mu$ the field strength of the U(1) gauge field A_μ, and $\Lambda = -3/l^2$ with l the AdS radius. The black hole solution reads

$$ds^2 = -f(r)dt^2 + \frac{dr^2}{f(r)} + r^2(d\theta^2 + \sin\theta^2 d\phi^2)\,, \tag{S2}$$

where

$$f(r) = 1 - \frac{2M}{r} + \frac{Q^2}{r^2} + \frac{r^2}{l^2}\,, \tag{S3}$$

and the gauge potential $A_t = -Q/r$. The parameter M represents the ADM mass of the black hole and Q the total charge.

The black hole event horizon r_h is determined as a larger root of $f(r_h) = 0$. The Hawking temperature and entropy are given by $T = \frac{1}{4\pi r_h}\left(1 + \frac{3r_h^2}{l^2} - \frac{Q^2}{r_h^2}\right)$ and $S = \pi r_h^2$, respectively. The chemical potential is given by $\mu = Q/r_h$, which measures the potential difference between the horizon and infinity. For example, one can obtain the specific heat $C_Q \equiv T\frac{\partial S}{\partial T}|_Q = \frac{2\pi r_h^2\left(-l^2Q^2 + l^2r_h^2 + 3r_h^4\right)}{3l^2Q^2 - l^2r_h^2 + 3r_h^4}$. Moreover, in the extended phase space, the thermodynamic volume reads $V = 4\pi r_h^3/3$ and the specific volume is given by $v = 2r_h$.

B. The RN black hole in AdS$_5$

The natural extension of the above-disuccsed black hole is to consider higher dimensions. To illustrate this, we investigate a five-dimensional spherical RN-AdS black hole as a representative case. The geometry is given by [1]

$$\begin{aligned} ds^2 &= -f(r)dt^2 + \frac{dr^2}{f(r)} + r^2 d\Omega_3^2\,, \\ f(r) &= 1 - \frac{8M}{3\pi r^2} + \frac{Q^2}{r^4} + \frac{r^2}{l^2}\,, \end{aligned} \tag{S4}$$

with $d\Omega_3^2$ the metric of 3-sphere. The thermodynamic pressure in the extended phase space is defined as

$$P = -\frac{\Lambda}{8\pi} = \frac{3}{4\pi l^2}\,. \tag{S5}$$

In the canonical ensemble, one has the first law of black hole thermodynamics:

$$dM = TdS + \mu dQ + VdP\,, \tag{S6}$$

where $V = \pi^2 r_h^4/2$ is the thermodynamic volume conjugate to P with r_h the location of the event horizon.

Using the 'Euclidean trick', one can obtain the black hole temperature

$$T = \frac{f'(r_h)}{4\pi} = \frac{1}{2\pi r_h}\left(1 + \frac{2r_h^2}{l^2} - \frac{Q^2}{r_h^4}\right), \tag{S7}$$

and the pressure

$$P = \frac{3}{8\pi r_h^2}\left(2\pi T r_h - 1 + \frac{Q^2}{r_h^4}\right). \tag{S8}$$

By identifying the specific volume as $v = 2r_h$, one obtains the equation of state

$$P = \frac{3T}{2v} - \frac{3}{2\pi v^2} + \frac{24Q^2}{\pi v^6}. \tag{S9}$$

It closely resembles the 4-dimensional RN black hole, except for the power of the last term in v.

The critical point is determined by

$$\left(\frac{\partial P}{\partial v}\right)_{T,Q} = 0, \quad \left(\frac{\partial^2 P}{\partial v^2}\right)_{T,Q} = 0\,, \tag{S10}$$

from which one obtains

$$T_c = \frac{2\sqrt{2}}{5\sqrt[4]{15}\pi\sqrt[4]{Q^2}},\; v_c = (60Q^2)^{1/4},\; P_c = \frac{1}{6\sqrt{15}\pi Q}\,. \tag{S11}$$

To compute the critical exponents for the critical point, we introduce

$$t = \frac{T - T_c}{T_c}, \quad \omega = \frac{v - v_c}{v_c}\,. \tag{S12}$$

This notation is not to be confused with the temporal coordinate t. Then one obtains the equation of state near the critical point:

$$\hat{P} \equiv \frac{P}{P_c} = 1 + \frac{12}{5}t - \frac{12}{5}t\omega - 2\omega^3 + O(\omega^4, t\omega^2)\,. \tag{S13}$$

We are interested in the following two critical exponents. The critical exponent β describes the behavior of the order parameter:

$$\eta = v_l - v_s \propto |t|^\beta\,. \tag{S14}$$

with v_s and v_l the specific volumes for small and large black holes. The second critical exponent γ describes the divergence of isothermal compressibility:

$$\kappa_T = -\frac{1}{v}\left(\frac{\partial v}{\partial P}\right)_T \propto |t|^{-\gamma}\,. \tag{S15}$$

For an isotherm at temperature $t < 0$, there is a first-order phase transition. At the transition point, both phases possess the same Gibbs free energy, in accordance with Maxwell's equal-area rule:

$$\oint v dP = 0\,, \tag{S16}$$

which replaces the 'oscillating part' of the isotherm by an isobar. Applying (S16) for fixed $t < 0$, one has

$$\int_{\omega_l}^{\omega_s} \omega \left(\frac{\partial \hat{P}}{\partial \omega}\right)_t d\omega = \int_{\omega_l}^{\omega_s} \omega \left(-\frac{12}{5}t - 6\omega^2\right) d\omega = 0\,, \tag{S17}$$

from which

$$\left[-\frac{6}{5}t\omega^2 - \frac{3}{2}\omega^4\right]_{\omega_l}^{\omega_s} = 0\,. \tag{S18}$$

Moreover, during the phase transition the pressure remains constant:

$$\hat{P}(\omega_s) = \hat{P}(\omega_l)\,. \tag{S19}$$

Then from (S13) one has

$$-\frac{12}{5}t(\omega_s - \omega_l) - 2(\omega_s^3 - \omega_l^3) = 0\,. \tag{S20}$$

The two equations (S18) and (S20) have a unique non-trivial solution $\omega_s = -\omega_l = \sqrt{6/5}\sqrt{-t}$. Therefore, one has the order parameter given by

$$\eta = v_c(\omega_l - \omega_s) = 2v_c\sqrt{-\frac{6}{5}t} \propto \sqrt{-t} \Rightarrow \beta = \frac{1}{2}\,. \tag{S21}$$

Moreover, from (S13), one has

$$\left(\frac{\partial \hat{P}}{\partial \omega}\right)_t = -\frac{12}{5}t - 6\omega^2\,. \tag{S22}$$

Then, along the critical isochore ($\omega = 0$), it becomes

$$\left(\frac{\partial \hat{P}}{\partial \omega}\right)_t = -\frac{12}{5}t \quad \Rightarrow \quad \left(\frac{\partial \omega}{\partial \hat{P}}\right)_t = -\frac{5}{12t}\,. \tag{S23}$$

Hence, the isothermal compressibility scales as

$$\kappa_T \propto \left(\frac{\partial v}{\partial P}\right)_T = \frac{v_c}{P_c}\left(\frac{\partial \omega}{\partial \hat{P}}\right)_t \propto \frac{1}{t} \Rightarrow \gamma = 1\,. \tag{S24}$$

C. Hairy electrically charged black hole

This model is an exact hairy black hole constructed by RN-AdS model with a non-trivial self-interacting scalar potential. The action is given by [2]:

$$\begin{aligned} I\left[g_{\mu\nu}, A_\mu, \phi\right] = \frac{1}{16\pi}\int_{\mathcal{M}} d^4x\sqrt{-g}\,\Big[R - e^{\phi}F_{\mu\nu}F^{\mu\nu} \\ -\frac{1}{2}\left(\partial\phi\right)^2 - V(\phi)\Big]\,, \end{aligned} \tag{S25}$$

where the dilaton potential takes the form:

$$V(\phi) = \left(\frac{\Lambda}{3} + \phi\right)(4 + 2\cosh\phi) - 6\sinh\phi\,. \tag{S26}$$

The hairy black hole solution is given by

$$ds^2 = \Omega(x)\left[-f(x)dt^2 + \frac{\eta^2 dx^2}{x^2 f(x)} + d\theta^2 + \sin^2\theta d\phi^2\right], \tag{S27}$$

$$A_t = \left(\frac{q}{x} - C\right), \quad \phi = \ln(x)\,, \tag{S28}$$

where C is a constant and the explicit expressions for the conformal factor $\Omega(x)$ and metric function $f(x)$ are

$$\begin{aligned} \Omega(x) &= \frac{x}{\zeta^2(x-1)^2}\,, \\ f(x) &= \frac{x^2-1}{2x} - \ln x + \frac{\zeta^2(x-1)^2}{x}\left(1 - \frac{2q^2}{x}\right) - \frac{\Lambda}{3}\,. \end{aligned} \tag{S29}$$

At the event horizon x_h, one has $f(x_h) = 0$. The constant C in the gauge potential is fixed by $A_t(x_h) = 0$. We focus on the branch that corresponds to thermodynamically stable black holes in flat space in the limit $\Lambda = 0$.

In the extended framework with $P = -\Lambda/(8\pi)$, As shown in [2], the system in the *canonical ensemble* (fixed charge Q) shows *two distinct critical points*, unlike the single critical point in the RN-AdS case. We now derive the critical exponents β and γ for the hairy black holes in the canonical ensemble.

The temperature is given by

$$T = \frac{(x_h-1)^2}{8\pi\zeta x_h}\left[-1 - 2\zeta^2\left(\frac{x_h+1}{x_h-1}\right) + 4\zeta^4 Q^2\left(\frac{x_h+2}{x_h}\right)\right], \tag{S30}$$

and the specific volume reads

$$v = \frac{1}{\zeta}\left(\frac{x_h+1}{x_h-1}\right)\,. \tag{S31}$$

with the parameter ζ determined from the horizon equation

$$\zeta = \frac{1}{2Q}\sqrt{\frac{x_h}{x_h-1}}\left[1 + \sqrt{1 + \frac{4(16\pi x_h P - 6x_h\ln x_h + 3x_h^2 - 3)Q^2}{3x_h(x_h-1)}}\right]^{1/2}\,. \tag{S32}$$

Unlike the AdS-RN black hole, it is not possible to get an analytic expression $P = P(v, T, Q)$ for the hairy case. Nevertheless, one has the same criticality conditions (S10). There are two critical points for any value of Q, including a RN-AdS–like critical point at large volume and low temperature, and another critical point at small volume and high temperature. As Q is varied, the critical compressibility factor $Z_c = P_c v_c/T_c$ changes continuously, effectively tuning the nature of the fluid in dual theory.

For a fixed Q, we introduce the expansion variables t and ω near each critical point, following the definition given in (S12). Near the critical point, the equation of state can then be expanded as:

$$P = P_c + At + Bt\omega + C\omega^3 + \mathcal{O}(t\omega^2, \omega^4)\,, \tag{S33}$$

following from the criticality conditions (S10). Here the parameters A, B, C depend solely on Q.

Differentiating equation (S33) at fixed $t < 0$, one has

$$dP = Btd\omega + 3C\omega^2 d\omega\,. \tag{S34}$$

Let ω_s and ω_l denote the specific volume deviations for small and large black holes, respectively. The pressure equality condition gives

$$P_c + At + Bt\omega_s + C\omega_s^3 = P_c + At + Bt\omega_l + C\omega_l^3\,, \tag{S35}$$

which simplifies to

$$Bt(\omega_s - \omega_l) + C(\omega_s^3 - \omega_l^3) = 0\,. \tag{S36}$$

Note that the Maxwell's equal area law (S16) requires

$$\int_{\omega_s}^{\omega_l} \omega(Bt + 3C\omega^2)d\omega = 0\,. \tag{S37}$$

By solving equations (S36) and (S37), we have $\omega_l = -\omega_s = \omega_0$. Then one obtains from (S36) that

$$Bt + C\omega_0^2 = 0\,, \quad \Rightarrow \quad \omega_0^2 = -\frac{B}{C}t\,. \tag{S38}$$

The order parameter (volume difference between phases) is given as

$$\eta = v_c(\omega_l - \omega_s) = 2v_c\sqrt{-\frac{B}{C}}\sqrt{-t} \propto \sqrt{-t} \Rightarrow \beta = \frac{1}{2}\,. \tag{S39}$$

Furthermore, from (S33), the pressure derivative is

$$\left(\frac{\partial P}{\partial v}\right)_T = Bt + 3C\omega^2\,. \tag{S40}$$

Near the critical point ($\omega \to 0$), one has

$$\left(\frac{\partial P}{\partial v}\right)_T \approx Bt\,. \tag{S41}$$

Thus, the isothermal compressibility behaves as

$$\kappa_T = -\frac{1}{v}\left(\frac{\partial v}{\partial P}\right)_T \approx -\frac{1}{v_c}\cdot\frac{1}{Bt} \propto \frac{1}{t} \Rightarrow \gamma = 1\,. \tag{S42}$$

D. Gauss-Bonnet Black Hole

We consider the Gauss–Bonnet black hole in AdS_5 space, incorporating higher-curvature corrections. The action in five-dimensional spacetime is given by [3]:

$$S = \frac{1}{16\pi}\int d^5x\sqrt{-g}\left[R - 2\Lambda + \alpha_{\rm GB}R_{\rm GB} - F_{\mu\nu}F^{\mu\nu}\right], \tag{S43}$$

where $R_{\rm GB} = R_{\mu\nu\gamma\delta}R^{\mu\nu\gamma\delta} - 4R_{\mu\nu}R^{\mu\nu} + R^2$. The charged black hole with spherical symmetric horizon reads

$$ds^2 = -f(r)dt^2 + f^{-1}(r)dr^2 + r^2 d\Omega_3^2\,, \tag{S44}$$

where

$$f(r) = 1 + \frac{r^2}{4\alpha_{\rm GB}}\left(1 - \sqrt{1 + \frac{64\alpha_{\rm GB}M}{3\pi r^4} - \frac{2\alpha_{\rm GB}Q^2}{3r^6} - \frac{32\pi\alpha_{\rm GB}P}{3}}\right), \tag{S45}$$

and from which the thermodynamic quantities are

$$M = \frac{\pi}{8}\left(4\pi P r_h^4 + 3r_h^2 + 6\alpha_{\rm GB} + \frac{Q^2}{4\pi r_h^2}\right), \tag{S46}$$

$$T = \frac{8\pi P r_h^3 + 3r_h - \frac{Q^2}{8\pi r_h^3}}{2\pi(r_h^3 + 8\alpha_{\rm GB} r_h)}, \tag{S47}$$

$$S = \frac{\pi^2}{2}\left(r_h^3 + 12\alpha_{\rm GB} r_h\right), \tag{S48}$$

$$P = -\frac{\Lambda}{8\pi} = \frac{3}{4r_h}\left(1 + \frac{4\alpha_{\rm GB}}{r_h^2}\right)T - \frac{3}{8\pi r_h^2} + \frac{Q^2}{8\pi r_h^6}. \tag{S49}$$

This model exhibits Van der Waals-type P–V criticality and small/large black hole phase transitions. Following a computational strategy similar to that used in previous sections, we obtain the critical exponents $\beta = \frac{1}{2}$ and $\gamma = 1$.

E. Charged black hole in the Barrow model

The Barrow model incorporates quantum gravitational effects through a quasi-fractal structure of spacetime, often referred to as spacetime foam. In this approach, the black hole entropy is modified according to the relation proposed by Barrow [4],

$$S_B = \left(\frac{A}{4G}\right)^{1+\delta/2}, \tag{S50}$$

where δ is the Barrow parameter ($0 \le \delta \le 1$) quantifying the deviation from a smooth horizon geometry. Here $\delta = 0$ corresponds to the standard Bekenstein–Hawking entropy, and $\delta = 1$ corresponds to the maximal fractal influence.

Within this framework, the metric for a charged AdS black hole is given by (S2). Nevertheless, the thermodynamic quantities are modified by the Barrow parameter δ:

$$S = \left(\pi r_h^2\right)^{1+\frac{\delta}{2}}, \tag{S51}$$

$$T = \frac{8\pi P r_h^4 + r_h^2 - Q^2}{2\pi^{1+\frac{\delta}{2}}(\delta+2) r_h^{3+\delta}}, \tag{S52}$$

$$P = \frac{3}{8\pi\ell^2} = \frac{(1+\delta)^2\left(\pi r_h^2\right)^{\delta/2} T}{4r_h} + \frac{Q^2}{8\pi r_h^4} - \frac{1}{8\pi r_h^2}, \tag{S53}$$

$$V = \frac{4}{3}\pi r_h^3. \tag{S54}$$

The modified Smarr relation becomes

$$M = (2+\delta)TS + \mu Q - 2PV. \tag{S55}$$

Adopting a similar computational strategy, we calculate the critical exponents, yielding $\beta = \frac{1}{2}$ and $\gamma = 1$.

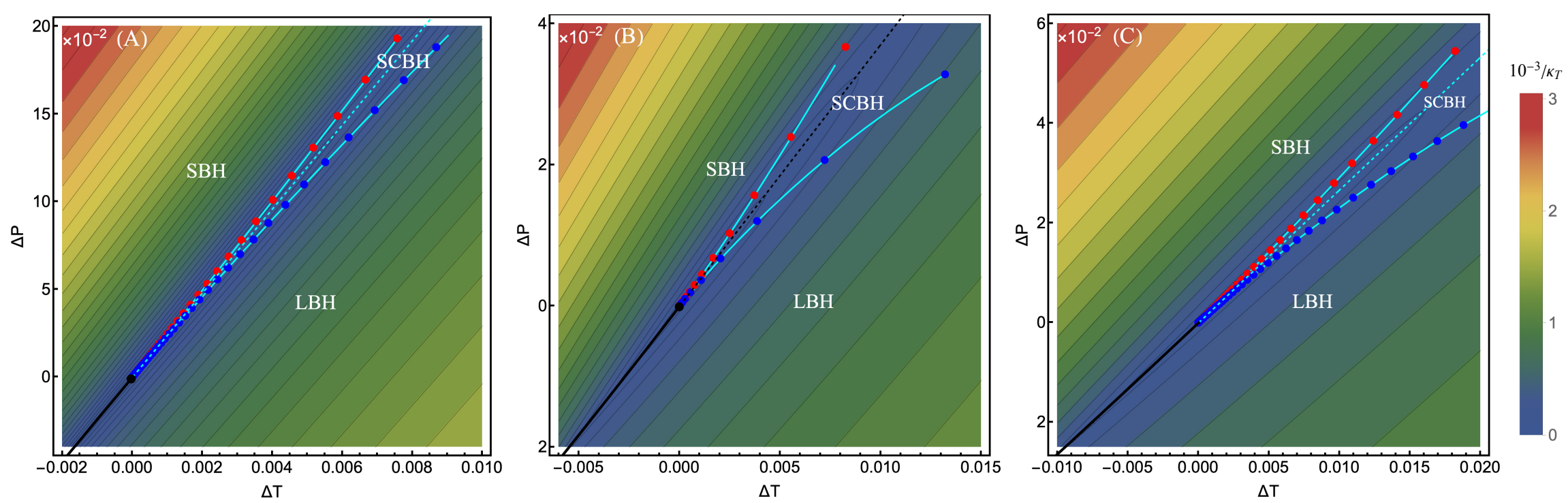

Fig. S1: Phase diagrams corresponding to (A) RN-AdS$_5$ black hole, (B) Gauss-Bonnet black hole in AdS$_5$ space, and (C) Charged AdS Black Hole of Barrow model. In all three systems, the charge is set to $Q = 1$. For the Gauss-Bonnet black hole we set $\alpha_{\rm GB} = 1/2$, and for the Barrow-model black hole we set $\delta = 0.8$.

F. Supercritical crossover lines $L^{\pm}$

For each model, we have derived the critical exponents β and γ at their respective critical points, confirming their adherence to mean-field values. Subsequently, we numerically determine the supercritical crossover lines $L^{\pm}$ following the methodology outlined in the End Matter. The scaling behaviors of these lines, governed by Eqs. (2) and (3) in the main text, are fully verified. The results, as exemplified in Fig. 4 of main text for selected critical points (see Fig. S1 for phase diagrams), demonstrate that the proposed scalings hold universally across these diverse and complex black hole backgrounds, reinforcing the mean-field nature of their criticality and the generality of our findings. We point out that the hairy electrically charged black hole with two critical points are clearly beyond the mean-field Van der Waals equation of state.

S2 EOSs and phase diagrams of the LGPT and LLPT

To study the supercritical thermodynamics near a LGPT, we collect the EOS of water, $\hat{P}(\hat{\rho};\hat{T})$, in the supercritical regime from the NIST database [5]. The thermodynamic variables are rescaled by their critical values, including temperature $\hat{T} = T/T_{\rm c}$, pressure $\hat{P} = P/P_{\rm c}$ and density $\hat{\rho} = \rho/\rho_{\rm c}$, where $T_{\rm c} = 647.10$ K, $P_{\rm c} = 22.06$ MPa and $\rho_{\rm c} = 17.87$ mol/l. The pressure P, temperature T, and density ρ are regarded respectively as the external field, control field, and order parameter (see Table 1 in main text). The corresponding response function is the susceptibility (compressibility) $\beta_T = \frac{P_{\rm c}}{\rho_{\rm c}} \frac{\partial \rho}{\partial P}\Big|_T$. The $L^{\pm}$ lines are determined by finding the maximum β_T along paths parallel to the critical isochore. Fig. S2(A,B) shows typical EOSs and the phase diagram near the LGPT, to be compared with those for 4D RN-Ads black holes in the extended phase space (Fig. 1A,B in main text).

To study the supercritical thermodynamics near a LLPT, we consider a generic mean-field two-state liquid-liquid model [6, 7] (see Sec. S3A for details). For a given system, the model can be used to fit the experimental data. The model parameters are taken from [7] such that the EOS given by the model agrees quantitatively with the available experimental data of supercooled water near the conjectured low-density liquid (LDL) to high-density liquid (HDL) phase transitions and the associated critical point. As summarized in Table 1 of main text, the order parameter of the LLPT is S, since the transition is entropy-driven. The typical EOSs and the phase diagram of the two-state model are plotted in Fig. S2C,D, to be compared with those for 4D RN-Ads black holes

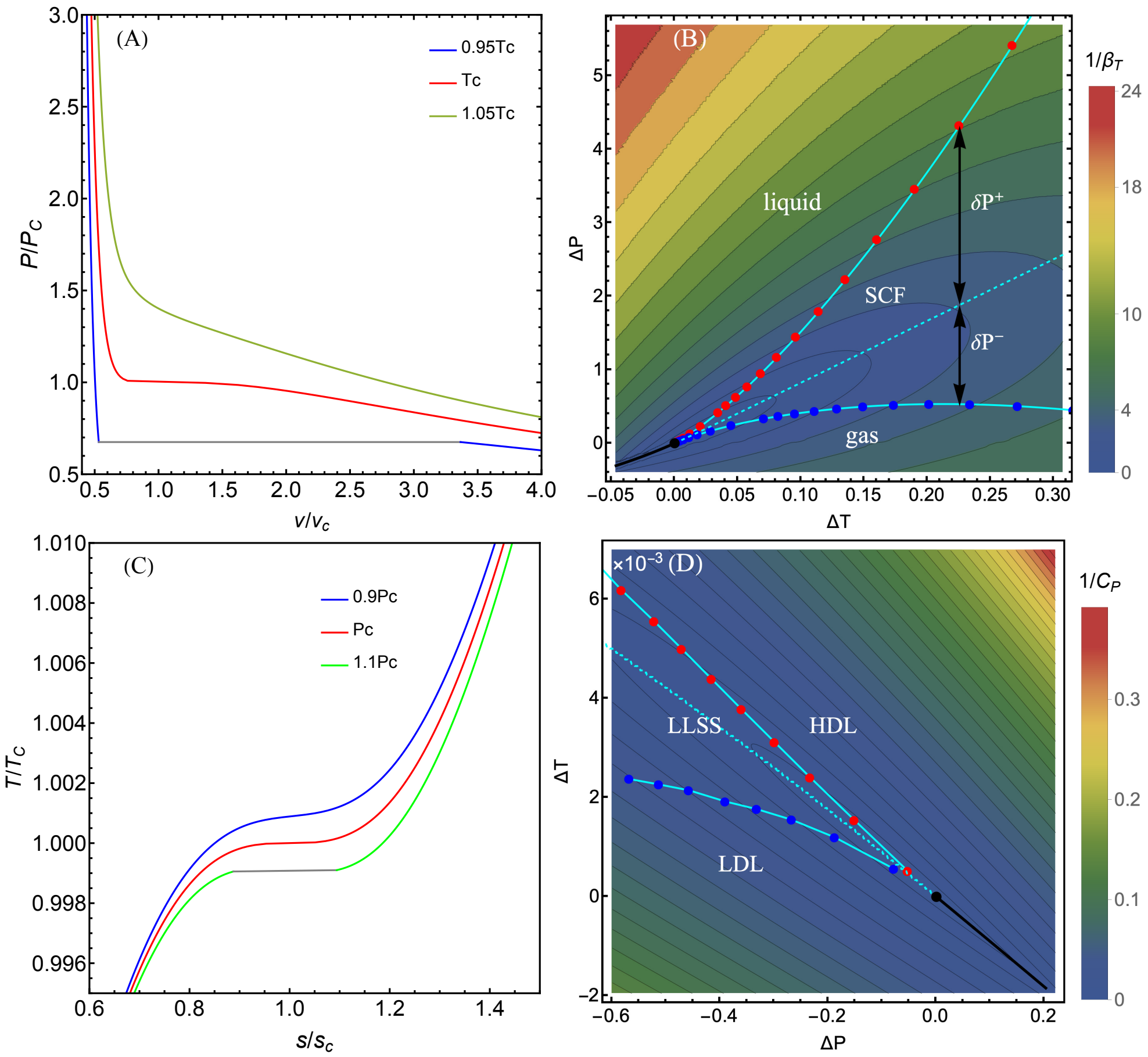

Fig. S2: (A) EOSs and (B) supercritical phase diagram of the LGPT in water. Data are obtained from the NIST database. (C) EOSs and (D) supercritical phase diagram of the LLPT in the two-sate water model [6, 7] (see Sec. S3A).

in the non-extended phase space (Fig. 2 in main text). Since the existence of LLPTs in water is still under debate [8], we further analyze a linear scaling theory with a negatively sloped ($dP/dT < 0$) coexistence line, which is a well-known theoretical model for general LLPTs (see Sec. S3B) [9]. The robustness of the universal supercritical scalings is confirmed.

S3 Liquid-liquid phase transitions

To investigate the supercritical thermodynamics for liquid-liquid phase transitions (LLPTs), we first study a concrete two-state model of supercooled water. Then we discuss a linear scaling theory with a negative slope coexistence line, which can be considered as a general theoretical description of LLPTs.

A. Two-state model of supercooled water

The two-state model describes the thermodynamics of a polyamorphic single component liquid, which can be regarded as a "mixture" of two interconvertible states, A and B, with concentrations $1 - x$ and x. In water, the two states A and B correspond respectively to the high density liquid

(HDL) and low density liquid (LDL) [10]. We consider the mean-field version developed in Refs. [6, 7].

The molar Gibbs free energy of the mixture solution reads,

$$\frac{G}{k_\mathrm{B}T} = \frac{G^\mathrm{A}}{k_\mathrm{B}T} + x\frac{G^\mathrm{BA}}{k_\mathrm{B}T} + x\ln x + (1-x)\ln(1-x) + \omega x(1-x), \tag{S56}$$

where k_B is the Boltzmann constant, $x\ln x + (1-x)\ln(1-x)$ the mixing entropy, $\omega x(1-x)$ the excess entropy of mixing due to interactions between A and B, and $\omega = 2 + \omega_0 \Delta P$ with ω_0 a fitting parameter. The term $G^\mathrm{A} = \sum_{m,n} c_{mn}(\Delta T)^m(\Delta P)^n$ is the Gibbs free energy of the pure state A, which can be determined by fitting experimental data with c_{mn} as adjustable coefficients. The term $G^\mathrm{BA} = G^\mathrm{B} - G^\mathrm{A}$ is the Gibbs free energy difference between A and B. Near the LLPTs, one assumes that

$$G^\mathrm{BA}/k_\mathrm{B}T = \lambda(\Delta T + a\Delta P + b\Delta T\Delta P)\,, \tag{S57}$$

where λ, a, b are fitting parameters. At the LLPT (on the coexistence line), $G^\mathrm{BA} = 0$, from which one can see that the parameters a and b capture the slope and curvature of the coexistence line. The fraction x is determined by the equilibrium condition

$$\begin{aligned}\mu^\mathrm{BA} &= \left(\frac{\partial G}{\partial x}\right)_{T,P} \\ &= G^\mathrm{BA} + k_\mathrm{B}T\left[\ln\frac{x}{1-x} + \omega(1-2x)\right] = 0\,,\end{aligned} \tag{S58}$$

whose solution is plugged into Eq. (S56). The critical values T_c, ρ_c and P_c are also fitting parameters. It can be shown that this model obey critical scalings with mean-field exponents.

With the expressions above, the EOSs can be derived. For example, $1/\hat\rho(P,T) = \hat V(P,T) = \left(\frac{\partial \hat G}{\partial \hat P}\right)_T$ and $\hat S(P,T) = -\left(\frac{\partial \hat G}{\partial \hat T}\right)_P$ using Eq. (S56). In Ref. [7], the model EOSs are fitted to the published experimental data in the range of 140 K to 310 K and 0.1 MPa to 400 Mpa. The best fitting gives $T_\mathrm{c} = 227.42$ K, $P_\mathrm{c} = 13.45$ MPa. Other fitting parameters can be found in [7]. The resulting EOSs are plotted in Fig. S2C. The $L^\pm$ lines are determined by finding the maxima of C_P along paths parallel to the critical isentrope (the line of $\hat S = \hat S_\mathrm{c}$, where $\hat S_\mathrm{c}$ is the entropy at the critical point).

B. Linear scaling theory

Near a critical point, the thermodynamic potential $\Psi(h_1, h_2)$ obeys a homogeneous scaling form

$$\Psi(h_1,h_2) = |h_2|^{2-\alpha}\, f\big(h_1/|h_2|^{\beta+\gamma}\big)\,, \tag{S59}$$

where h_1 and h_2 are the ordering and thermal scaling fields respectively, $f(x)$ is a universal scaling function, and α, β, γ are universal critical exponents related by the scaling law $\alpha + 2\beta + \gamma = 2$ [11].

Following Ref. [9], the scaling fields are taken as linear combinations of the physical fileds P and T,

$$\begin{aligned} h_1 &= \Delta P\cos\varphi - \Delta T\sin\varphi, \\ h_2 &= \Delta P\sin\varphi + \Delta T\cos\varphi, \end{aligned} \tag{S60}$$

with $\Delta P = P/P_\mathrm{c} - 1$, $\Delta T = T/T_\mathrm{c} - 1$, and $\tan\varphi = d\hat P/d\hat T$ being the slope of the coexistence curve. For symmetric systems (e.g. the Ising or lattice-gas model) $\varphi = 0$, so that $h_1 = \Delta P$ and $h_2 = \Delta T$. Real liquid–gas or liquid–liquid phase transitions are generally asymmetric, $\varphi \neq 0$, and therefore the physical fields mix both h_1 and h_2.

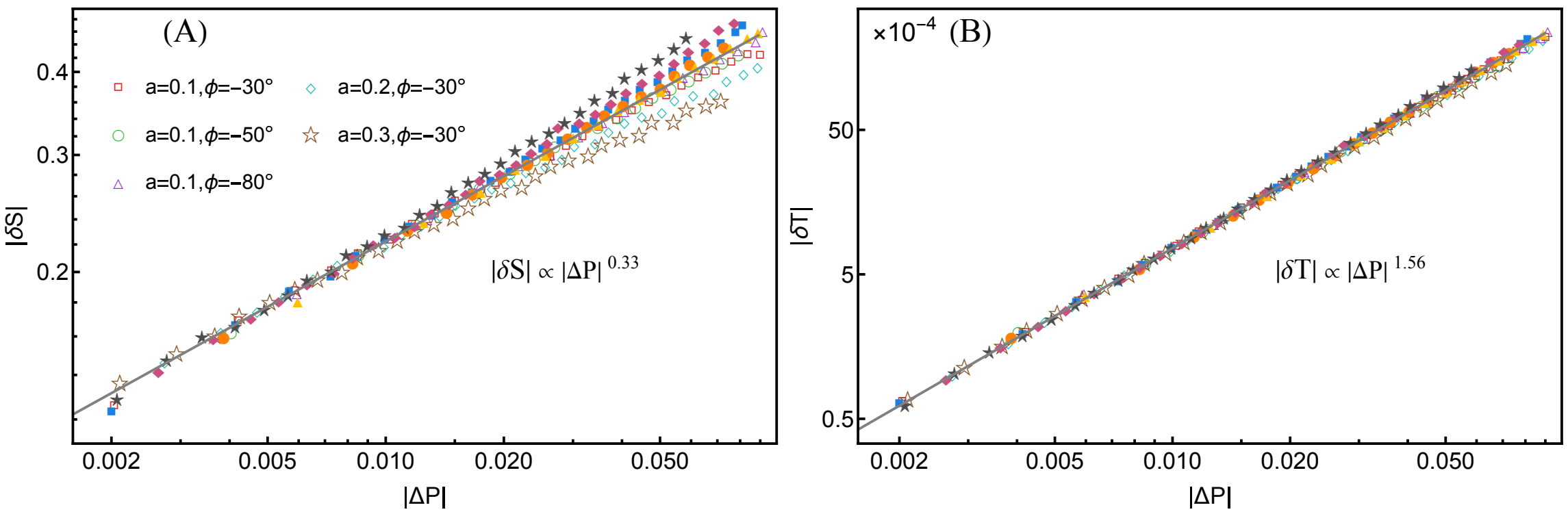

Fig. S3: Data of $L^{\pm}$ lines given by the linear scaling theory. (A) Scaling law between the reduced entropy (order parameter) ΔS and reduced pressure ΔP. (B) Scaling law between two reduced fields δT and ΔP.

A convenient parametric representation of the scaling fields is provided by the linear scaling theory [12, 13]. Introducing "polar" variables r (a measure of the distance from the critical point) and $\theta \in [-1, 1]$ (an angular variable, with $\theta = \pm 1$ on the coexistence curve), one writes

$$\begin{aligned} h_1 &= a\, r^{\beta\delta}\, \theta\, (1-\theta^2)\,, \\ h_2 &= r\,(1 - b^2\theta^2)\,, \end{aligned} \tag{S61}$$

where δ is a critical exponent linked to β and γ by $(\delta-1)\beta = \gamma$, a is a non-universal constant, and b is a universal parameter given by $b^2 = \frac{\delta-3}{(\delta-1)(1-2\beta)}$. With this parametrisation, the thermodynamic potential becomes

$$\Psi(r,\theta) = r^{\beta(\delta+1)}\, p(\theta)\,, \tag{S62}$$

where $p(\theta)$ is an analytic function.

The order parameter conjugate to h_1 is,

$$\phi_1 = \left(\frac{\partial \Psi}{\partial h_1}\right)_{h_2} = r^{\beta}\, m(\theta)\,. \tag{S63}$$

In the linear scaling theory, one assumes $m(\theta) = k\,\theta$, with k another system-dependent parameter. The order parameter conjugate to h_2 is,

$$\phi_2 = \left(\frac{\partial \Psi}{\partial h_2}\right)_{h_1} = r^{\beta(\delta+1)-1}\, s(\theta)\,. \tag{S64}$$

The function $s(\theta)$ is determined from the Maxwell relation $\left(\partial\phi_1/\partial h_2\right)_{h_1} = \left(\partial\phi_2/\partial h_1\right)_{h_2}$, which yields

$$s(\theta) = ak\big(s_0 + s_2\theta^2\big)\,, \tag{S65}$$

with the coefficients

$$\begin{aligned} s_0 &= -\frac{\beta\Big[-3+\delta+b^2(-1+\delta)(-2+\beta+\beta\delta)\Big]}{2b^4(-2+\beta+\beta\delta)(-1+\beta+\beta\delta)}\,, \\ s_2 &= \frac{\beta(\delta-3)}{2b^2(-2+\beta+\beta\delta)}\,. \end{aligned} \tag{S66}$$

The input to the theory consists of the universal critical exponents (β, γ, δ, etc.) and three system-specific parameters: the coexistence-line slope $\tan\varphi$, and the two constants a and k. Once

these parameters are fixed, all thermodynamic quantities are expressed through the two independent variables r and θ. For example, the physical fields are,

$$\begin{aligned}\Delta P &= a r^{\beta+\gamma}\,\theta(1-\theta^2)\cos\varphi + r(1-b^2\theta^2)\sin\varphi\,,\\ \Delta T &= r(1-b^2\theta^2)\cos\varphi - a r^{\beta+\gamma}\,\theta(1-\theta^2)\sin\varphi\,.\end{aligned} \tag{S67}$$

The reduced volume and reduced entropy are,

$$\Delta V = \left(\frac{\partial \Psi}{\partial \Delta P}\right)_{\Delta T} = -\phi_1\cos\varphi - \phi_2\sin\varphi\,, \tag{S68}$$

$$\Delta S = -\left(\frac{\partial \Psi}{\partial \Delta T}\right)_{\Delta P} = -\phi_1\sin\varphi + \phi_2\cos\varphi\,. \tag{S69}$$

The susceptibilities with respect to the scaling fields read

$$\begin{aligned}\chi_1 &\equiv \left(\frac{\partial \phi_1}{\partial h_1}\right)_{h_2} = \frac{k}{a}\,r^{-\gamma}\,C_1(\theta)\,,\\ \chi_2 &\equiv \left(\frac{\partial \phi_2}{\partial h_2}\right)_{h_1} = r^{-\alpha}\,C_2(\theta)\,,\\ \chi_{12} &\equiv \left(\frac{\partial \phi_1}{\partial h_2}\right)_{h_1} = \left(\frac{\partial \phi_2}{\partial h_1}\right)_{h_2} = k\,r^{\beta-1}\,C_{12}(\theta)\,,\end{aligned} \tag{S70}$$

where

$$\begin{aligned}C_1(\theta) &= \frac{1-b^2\theta^2(1-2\beta)}{C_0(\theta)}\,,\\ C_2(\theta) &= \frac{(1-\alpha)(1-3\theta^2)s(\theta) - 2s_2\beta\delta\theta^2(1-\theta^2)}{C_0(\theta)}\,,\\ C_{12}(\theta) &= \frac{\beta\theta\big[1-\delta-\theta^2(3-\delta)\big]}{C_0(\theta)}\,,\\ C_0(\theta) &= (1-3\theta^2)(1-b^2\theta^2) + 2\beta\delta b^2\theta^2(1-\theta^2)\,.\end{aligned} \tag{S71}$$

Physical response functions are linear combinations of χ_1, χ_2 and χ_{12}, weighted by the mixing angle φ:

$$\begin{aligned}\kappa_T &\equiv \left(\frac{\partial \Delta\rho}{\partial \Delta P}\right)_{\Delta T} = \chi_1\cos^2\varphi + \chi_{12}\sin 2\varphi + \chi_2\sin^2\varphi\,,\\ C_P &\equiv \left(\frac{\partial \Delta S}{\partial \Delta T}\right)_{\Delta P} = \chi_1\sin^2\varphi - \chi_{12}\sin 2\varphi + \chi_2\cos^2\varphi\,,\\ \alpha_P &\equiv \left(\frac{\partial \Delta V}{\partial \Delta T}\right)_{\Delta P} = \frac{1}{2}\Big[(\chi_1-\chi_2)\sin 2\varphi - 2\chi_{12}\cos 2\varphi\Big]\,.\end{aligned} \tag{S72}$$

The LLPT is characterized within the linear scaling theory framework, by a negative coexistence-line slope ($\tan\varphi < 0$) [9]. Critical exponents are taken from the 3D Ising universality class ($\beta \simeq 0.3265$, $\gamma \simeq 1.237$) [14]. Five sets of parameters are explored: $\varphi = -30°$ with $a = k = 0.1$, 0.2, and 0.3; $\varphi = -50°$ with $a = k = 0.1$; and $\varphi = -80°$ with $a = k = 0.1$. The $L^{\pm}$ lines computed from these sets yield the data shown in Fig. S3. Here we also choose the entropy to be the order parameter. The $L^{\pm}$ lines are then determined from the extrema of the heat capacity along paths parallel to the critical isentrope. The results provide complementary verification of the scaling laws of $L^{\pm}$ in LLPTs, thereby strengthening the evidence for its universality and reliability.

References

1. Niu C, Tian Y, and Wu XN. Critical Phenomena and Thermodynamic Geometry of RN-AdS Black Holes. Phys. Rev. D 2012;85:024017.
2. Astefanesei D, Mann RB, and Rojas R. Hairy Black Hole Chemistry. JHEP 2019;11:043.
3. Cai RG, Cao LM, Li L, and Yang RQ. P-V criticality in the extended phase space of Gauss-Bonnet black holes in AdS space. JHEP 2013;09:005.
4. Barrow JD. The Area of a Rough Black Hole. Phys. Lett. B 2020;808:135643.
5. NIST Chemistry WebBook. http://webbook.nist.gov/chemistry/.
6. Bertrand C and Anisimov M. Peculiar thermodynamics of the second critical point in supercooled water. The Journal of Physical Chemistry B 2011;115:14099–111.
7. Holten V and Anisimov MA. Entropy-driven liquid-liquid separation in supercooled water. Scientific Reports 2012;2, 713:713.
8. Gallo P, Amann-Winkel K, Angell CA, et al. Water: A tale of two liquids. Chemical reviews 2016;116:7463–500.
9. Luo J, Xu L, Lascaris E, Stanley HE, and Buldyrev SV. Behavior of the Widom Line in Critical Phenomena. Phys. Rev. Lett. 13 2014;112:135701.
10. Coronas LE and Franzese G. Phase behavior of metastable water from large-scale simulations of a quantitative accurate model: The liquid-liquid critical point. 2024.
11. Rushbrooke GS. On the Thermodynamics of the Critical Region for the Ising Problem. The Journal of Chemical Physics 1963;39:842–3.
12. Schofield P. Parametric Representation of the Equation of State Near A Critical Point. Phys. Rev. Lett. 12 1969;22:606–8.
13. Schofield P, Litster JD, and Ho JT. Correlation Between Critical Coefficients and Critical Exponents. Phys. Rev. Lett. 19 1969;23:1098–102.
14. Guida R and Zinn-Justin J. Critical exponents of the N-vector model. Journal of Physics A Mathematical General 1998;31:8103–21.
